# Ocular microvascular changes in patients with sepsis: a prospective observational study

**DOI:** 10.1186/s13613-020-00655-x

**Published:** 2020-04-07

**Authors:** Jurate Simkiene, Zivile Pranskuniene, Astra Vitkauskiene, Vidas Pilvinis, E. Christiaan Boerma, Andrius Pranskunas

**Affiliations:** 1 Department of Intensive Care Medicine, Lithuanian University of Health Sciences, Eiveniu str. 2, Kaunas, 50009 Lithuania; 2 Department of Drug Technology and Social Pharmacy, Lithuanian University of Health Sciences, Eiveniu str. 2, Kaunas, 50009 Lithuania; 3 Institute of Pharmaceutical Technologies, Lithuanian University of Health Sciences, Eiveniu str. 2, Kaunas, 50009 Lithuania; 4 Department of Laboratory Medicine, Lithuanian University of Health Sciences, Eiveniu str. 2, Kaunas, 50009 Lithuania; 5 Department of Intensive Care Medicine, Medical Center Leeuwarden, Henri Dunantweg 2, 8901 BR Leeuwarden, The Netherlands

**Keywords:** Conjunctival microcirculation, Retinal vessels, Fundus imaging, IDF imaging, Sepsis

## Abstract

**Background:**

The aim of the study was to detect differences in the conjunctival microcirculation between septic patients and healthy subjects and to evaluate the course of conjunctival and retinal microvasculature in survivors and non-survivors over a 24-h period of time.

**Methods:**

This single-center prospective observational study was performed in mixed ICU in a tertiary teaching hospital. We included patients with sepsis or septic shock within the first 24 h after ICU admission. Conjunctival imaging, using an IDF video microscope, and retinal imaging, using portable digital fundus camera, as well as systemic hemodynamic measurements, were performed at three time points: at baseline, 6 h and 24 h. Baseline conjunctival microcirculatory parameters were compared with healthy controls.

**Results:**

A total of 48 patients were included in the final assessment and analysis. Median APACHE II and SOFA scores were 16[12–21] and 10[7–12], respectively. Forty-four (92%) patients were in septic shock, 48 (100%) required mechanical ventilation. 19 (40%) patients were discharged alive from the intensive care unit. We found significant reductions in all microcirculatory parameters in the conjunctiva when comparing septic and healthy subjects. In addition, we observed a significant lower microvascular flow index (MFI) of small conjunctival vessels during all three time points in non-survivors compared with survivors. However, retinal arteriolar vessels were not different between survivors and non-survivors.

**Conclusions:**

Conjunctival microvascular blood flow was altered in septic patients. In the 24-h observation period conjunctival small vessels had a significantly higher MFI, but no difference in retinal arteriolar diameter in survivors in comparison with non-survivors.

*Trial registration* NCT04214743, https://www.clinicaltrials.gov. Date of registration: 31 December 2019 – Retrospectively registered, https://clinicaltrials.gov/ct2/show/NCT04214743

## Background

The eye is an organ site where the microcirculation can be observed directly in a non-invasive way and such provides a unique opportunity to study changes in the microvasculature in various disease states. Both conjunctival and retinal microvessels may provide useful information on onset, progression and prognosis of systemic diseases (e.g., diabetes, hypertension, coronary heart disease) and cerebral diseases (e.g., stroke, dementia) [1–7]. However, data on changes in sepsis are limited.

Sepsis is associated with a high mortality worldwide [8]. Microvascular alterations are considered a key characteristic of sepsis and its severity is related to morbidity and mortality [9–11]. In addition, microvascular alterations improved over 24 h after the onset of shock in response to therapy in survivors but not in non-survivors [12]. For practical reasons, changes of the human microcirculation during sepsis are predominantly evaluated in the sublingual region using handheld microscopes, including Sidestream Dark Field (SDF) imaging. Another easily accessible spot, such as the conjunctiva, has only been studied in animals during sepsis [13].

The retina and conjunctiva share a common origin of circulation, since the ophthalmic artery originates from the internal carotid artery and supplies both retina and conjunctiva. In this way, microvascular changes in retina and conjunctiva may be related to one another and both conjunctiva and retina could be regarded as an alternative opportunity for the evaluation of systemic sepsis-induced microcirculatory effects. In combination with the development of handheld digital fundus cameras for retinal imaging, direct observation of the retinal vessels is now also feasible at the bedside. In a previous study, we demonstrated differences in retinal vessels between healthy volunteers and septic patients [14].

The aim of this study was to detect potential differences in the conjunctival microcirculation between septic patients and healthy subjects and to evaluate the course of retinal and conjunctival microvascular changes in survivors and non-survivors over a 24-h period.

## Materials and methods

### Setting and patients

This single-center prospective observational study was performed between January 2018 and August 2019 in an 18-bed mixed ICU of a tertiary teaching hospital (The Hospital of Lithuanian University of Health Sciences). The study was approved by Kaunas Regional Biomedical Research Ethics Committee (Ethics approval number No. 2018/BE-2-18) and performed in compliance with the Helsinki Declaration. Written informed consent was obtained from the patients or next-of-kin, consistent with applicable laws. We registered this trial retrospectively with ClinicalTrials.gov under the identifier NCT04214743.

We included patients with sepsis or septic shock within the first 24 h after ICU admission [15]. Exclusion criteria were psychiatric disorders, brain diseases, chronic alcoholism, autoimmune rheumatic and various ophthalmological diseases, such as glaucoma, age-related macular degeneration, diabetic retinopathy, cataract and other ocular surface disorders (dry eyes, keratopathy, ocular infections).

Eye care in ICU patients with risk factors, such as mechanical ventilation and sedation, consists of manual closure of the eyes or lid taping along the lash margin, when any conjunctival or corneal exposure is visible. For all subjects, standard cleaning of the eyelids and surrounding skin with saline-soaked gauze was conducted twice daily. Eyes were examined daily for presence of conjunctival or corneal disorders. When eye damage occurred, an oculist was consulted. Before and after IDF imaging of conjunctiva, instillation of eye drops was performed. No other medications were used for ocular surface before IDF imaging of the conjunctiva.

Patients with sepsis and septic shock were managed according to the Surviving Sepsis Campaign guidelines [16]. Systemic hemodynamic were monitored invasively by measuring arterial blood pressure and other variables with a transpulmonary thermodilution device (PiCCO^®^, Pulsion Medical systems, Munich, Germany). Severity of illness according to acute physiology and chronic health evaluation (APACHE) II score was calculated over the first 24 h, and sequential organ failure assessment (SOFA) score was calculated on inclusion. Conjunctival and retinal imaging, as well as systemic hemodynamic measurements, were performed at three time points: at baseline, 6 h and 24 h. The control group consisted of 20 age-matched healthy volunteers with no reported ocular pathology, diabetes, non-controlled hypertension or any clinical eye-related complaint.

### Evaluation of the conjunctival microcirculation

Conjunctival microcirculation images were obtained using a Cytocam^®^-IDF device (Braedius Medical, Huizen, The Netherlands). This device is technically and optically optimized for visualization of the microcirculation on the surfaces of organs. IDF imaging is based on the principle that emitted green light (wavelength 530 nm) is absorbed by the hemoglobin content in red blood cells. Therefore, red blood cells are seen as black or gray bodies during imaging. The vessel walls are not visualized, so vessels can only be detected by the presence of red blood cells. A validation study showed that Cytocam-IDF imaging yielded better image quality than SDF imaging [17].

All conjunctival microcirculatory images were obtained in accordance with a local research protocol, selecting the nasal side of the conjunctival area. This allows steady imaging by carefully using the patient’s nose as a resting point for the hand of the camera operator. Selecting the same conjunctival area is also done to avoid large variations in microcirculatory parameters [18, 19].

We followed published expert recommendations for quality and analysis of obtained images [20].

Images from at least three areas were acquired and stored on a computer.

**Fig. 1:**
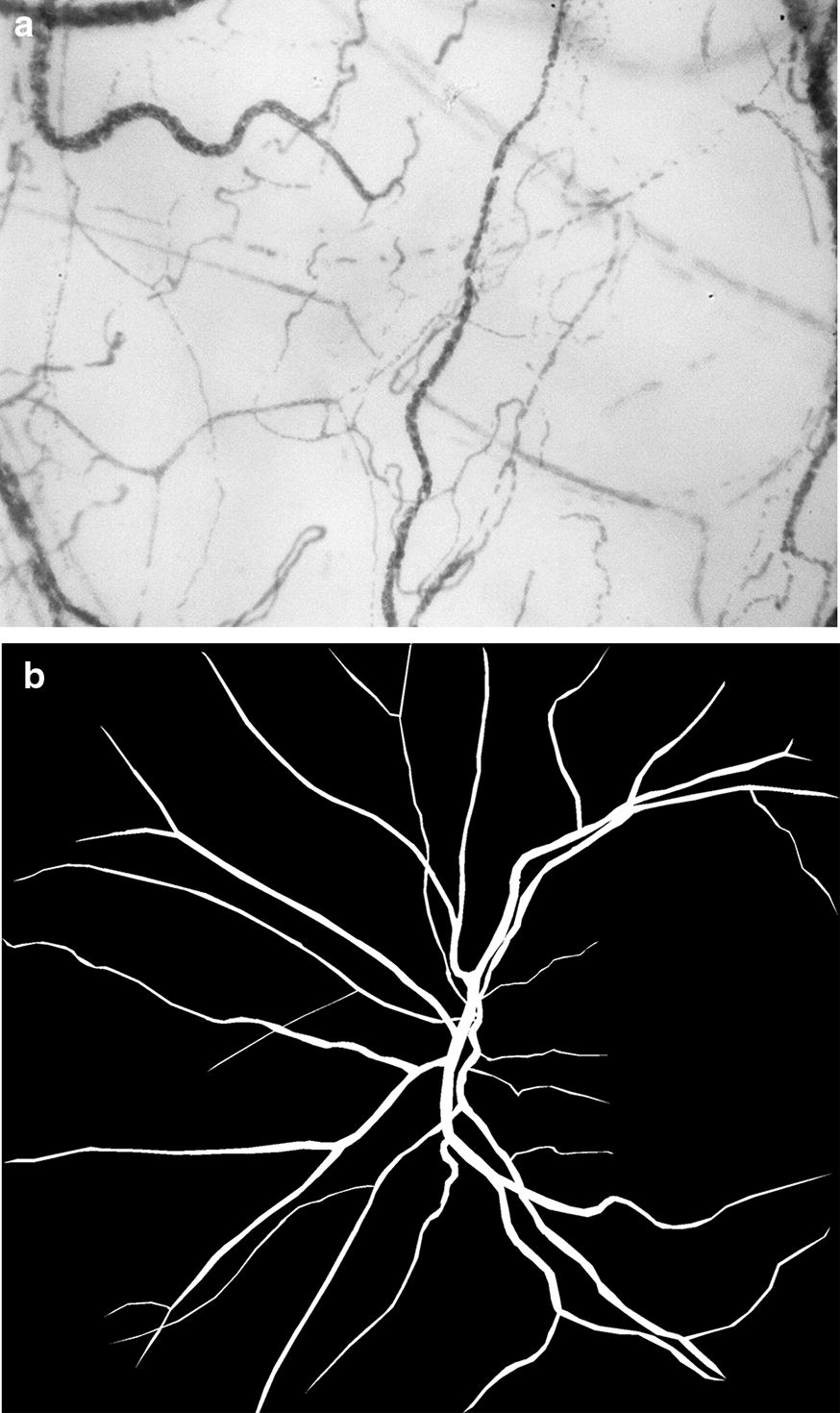
Images of ocular microvasculature at baseline in septic patient: **a** conjunctival microcirculation, **b** manually segmented retinal vascular networks of septic patient

Image clips were exported for analysis using validated AVA^®^ v3.0 software (Microvision Medical, Amsterdam, The Netherlands) [21]. Investigators analyzed the video clips offline in a blinded manner and in random order to prevent coupling. Each image was divided into four equal quadrants. Flow in small vessels was classified semi-quantitatively (no flow: 0; intermittent flow: 1; sluggish flow: 2; continuous flow: 3). We calculated the microvascular flow index (MFI) as the sum of each quadrant score divided by the number of quadrants in which the vessel type was visible [22]. We calculated the total vessel density (TVD) of small vessels using the AVA software package and a cut-off diameter for small vessels (mostly capillaries) of < 20 μm. Proportion of perfused vessels (PPV) of small vessels was computed by dividing the perfused small vessel length by the total length of small vessels. Perfused vessel density (PVD) of small vessels was calculated by measuring the density of perfused small vessels within the field of view (computed as proportion of perfused vessels multiplied by total vessel density). Images of ocular microvasculature at baseline in septic patient are presented in Fig. 1. Examples of conjunctival microcirculation are provided in the form of video files (Additional files 1, 2, 3).

### Fundus imaging and analysis

Twenty minutes after instillation of tropicamide (1%) color optic disc photographs were taken in full mydriasis, using an Optomed Aurora (Optomed Oy, Oulu, Finland) digital fundus camera (field of view 50 degrees). Measurements of vessel diameters were performed in the right eye. When the right eye was not feasible, the assessment was carried out in the left eye. Image analysis was performed using validated automatic retinal image analysis (ARIA) software [23]. At the end of the vessel analysis, ARIA produces a summary table with mean (± SD) diameter and length of each vessel within the analyzed area. Numbered vessels then were classified in arteries and veins [24].

Diameters were obtained from the largest six arterioles and venules coursing through an area of 0.5–1 disc diameter surrounding the optic disc (zone B). Based on the revised Knudtson–Parr–Hubbard formula, the average retinal arteriolar and venular caliber were calculated and summarized as the central retinal arteriolar equivalent (CRAE) and central retinal venular equivalent (CRVE) [25]. Manual segmentation was performed using “Vampire” software. (The Manual vessel segmentation tool is made available by the VAMPIRE group at vampire.computing.dundee.ac.uk.) The density of manually segmented retinal vessels was calculated using ImageJ software: vascular length density = skeletonized vessel area/total area * 100%.

### Statistics

Primary aims of the study were the differences in MFI in conjunctival small vessels between septic patients and healthy controls and differences between survivors and non-survivors. Secondary aim of the study was to detect differences in CRAE of retinal vessels between survivors and non-survivors. Considering detected means and standard deviations in pilot observations we estimated that a sample size should include at least 28 patients and 14 healthy controls (power 95%, alpha risk 5%) for all conjunctival microcirculatory parameters when comparing septic patients to healthy volunteers, and at least 12 patients in each group for MFI and PPV of small vessels (power 80%, alpha risk 5%) when comparing survivors to non-survivors.

Data were analyzed with Statistical Package for Social Sciences (SPSS 22 for Windows, Chicago, IL, USA). Quantitative variables’ distribution was tested with a Kolmogorov–Smirnov normality test. Since most of the parameters showed a non-normal distribution, data are presented as the median [interquartile range, IQR] and analyzed with non-parametric tests. Between groups differences were tested with a Mann–Whitney *U* test. Friedman test was done to assess the evolution of microvascular parameters in each group followed by a Wilcoxon test to evaluate intragroup evolution. Correlations were tested with a Spearman correlations test. The predictive value on outcome of initial microvascular parameters was calculated using a receiver operator characteristic (ROC) curve, and the area under the curve was computed. A *p* < 0.05 was considered significant.

## Results

### Population characteristics

During the study period 76 patients were screened. Fifty-eight patients met all inclusion criteria. Ten patients were excluded because of insufficient conjunctival or retinal image quality for the baseline assessment. Hence, a total of 48 patients were included in the final assessment and analysis. Median time to inclusion was 12 h from ICU admission. Baseline characteristics of included patients are shown in Table 1. Patients were predominantly male (63%) with a median age of 67 years [54–77], a median APACHE II score of 16[12–21] and a median SOFA score of 10[7–12]. Forty-four (92%) patients were in septic shock, 48 (100%) required mechanical ventilation. Nineteen patients (40%) were discharged alive from the intensive care unit.

**Table 1:** Baseline characteristics of the study group and comparison between ICU survivors versus non-survivors

	All patients (*n* = 48)	ICU Survivors (*n* = 19)	ICU Non-survivors (*n* = 29)
Age, years	67 [54–77]	58 [47–67]	75 [61–79]*
Male gender, *n* (%)	30 (63)	13 (68)	17 (59)
APACHE II score	16 [12–21]	13 [9–16]	18 [14–26]*
SOFA score at baseline	10 [7–12]	10 [6–11]	12 [9–13]*
Time after ICUadmission,* h*	12 [7–17]	12 [10–17]	13 [4–18]
Source of infection, *n* (%)			
Lung	22 (46)	11 (58)	11 (38)
Abdomen	17 (35)	5 (26)	12 (41)
Urinary tract	6 (13)	2 (11)	4 (14)
Soft tissue	2 (4)		2 (7)
Miscellaneous	1 (2)	1 (5)	
Heart rate, bpm	110 [86–131]	89 [77–116]	110 [86–131]
Mean arterial pressure, mmHg	73 [62–82]	77 [69–81]	73 [62–82]
Cardiac index, L/min/m^2^	3.0 [2.1–3.9]	2.9 [2.5–3.7]	3.0 [1.7–4.2]
Vasoactive drugs			
Norepinephrine, *n*, mcg/kg/min	44, 0.24 [0.13–0.35]	17, 0.24 [0.08–0.32]	27, 0.28 [0.16–0.40]
Epinephrine, *n*, mcg/kg/min	6, 0.09 [0.06–0.23]		6, 0.09 [0.06–0.23]
Dobutamine, *n*, mcg/kg/min	1, 6.0 [6.0–6.0]		1, 6.0 [6.0–6.0]
Hemoglobin, g/L	105 [90–124]	109 [98–135]	98 [82–114]*
White blood cells, *n *× 10^3^/mmc	13.9 [7.8–19.2]	13.4 [7.2–17.7]	14.0 [8.7–19.9]
CRP, mg/L	217 [151–282]	252 [139–324]	206 [161–257]
IL-6, pg/mL	1460 [511–4852]	1036 [459–4487]	1695 [511–4852]
IL-10, pg/mL	45 [9–135]	26 [8–220]	55 [11–154]
PaO_2_, mmHg	97 [80–135]	92 [82–110]	111 [71–148]
Arterial lactate, mmol/L	1.6 [1.1–3.6]	1.1 [0.9–1.5]	2.3 [1.5–4.9]*

Data are presented as median [IQR]

*APACHE* Acute Physiology and Chronic Health Evaluation, *SOFA* Sepsis-related Organ Failure Assessment, *ICU* intensive care unit, *CRP* c-reactive protein

*p < 0.05 between survivors and non-survivors

Survivors had lower APACHE II and SOFA scores, lower serum lactate concentrations, higher hemoglobin levels and were younger in comparison to non-survivors. We found no difference in the dose of initial vasopressor agents between survivors and non-survivors, and similar hemodynamic variables in both groups were observed at the time of inclusion (Table 1).

### Course of systemic hemodynamic parameters

We did not find a difference in mean arterial pressure between survivors and non-survivors during all three measurements. In non-survivors, heart rate was significant higher after 24 h (116[98–127] vs. 96[77–109 bpm, *p* = 0.019), noradrenaline dose was higher after 6 h 0.29[0.19–0.40] vs. 0.20[0.10–0.30] mcg/kg/min, *p* = 0.049) and 24 h (0.32[0.16–0.56] vs. 0.14[0.08–0.32] mcg/kg/min, *p* = 0.047); lactate levels were significant higher after 6 h (2.3[1.5–4.9] vs. 1.1[0.9–1.5] mmol/L, *p* < 0.001) and 24 h (1.6[1.3–3.3] vs. 1.2[0.9–1.6] mmol/L, *p* = 0.003) in comparison with survivors.

### Primary aim of the study: evaluation of the conjunctival microcirculation

**Fig. 2:**
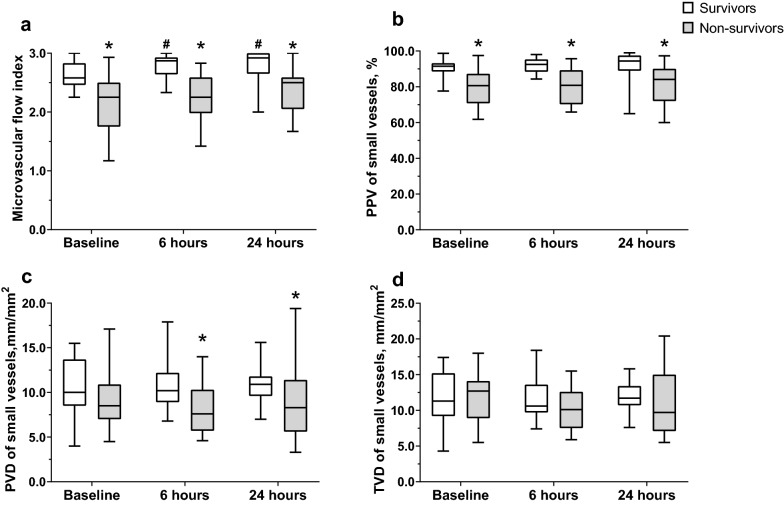
Box plots demonstrating the time course of microcirculatory perfusion parameters in survivors and non-survivors: **a** microvascular flow index; **b** proportion of perfused vessels (PPV) of small vessels; **c** perfused vessels density (PVD) of small vessels; **d** total vessel density (TVD) of small vessels. *p < 0.05 between survivors and non-survivors; ^#^*p* < 0.05 in comparison with baseline

We found significant reductions in all microcirculatory parameters in the conjunctiva of septic patients when compared to healthy subjects (Table 2). Throughout the observation, we found a significant lower MFI of small vessels during all three time points and significant lower PVD of small vessels at 6 and 24 h in non-survivors compared with survivors (Table 3, Fig. 2). There was no difference in TVD and De Backer score of small vessels between survivors and non-survivors during all three time points. In non-survivors, we observed no significant changes in microcirculatory parameters over time. However, survivors had a significantly improved MFI of small vessels at 6 and 24 h compared to baseline (Table 3, Fig. 2).

**Table 2:** Differences in microcirculation parameters of conjunctiva between septic patients and healthy subjects

Microcirculation parameter	Septic patients (*n* = 48)	Healthy control (*n* = 20)	*p*
TVD (small), mm/mm^2^	11.5 [9.2–14.4]	16.2 [13.2–21.9]	< 0.001
PVD (small), mm/mm^2^	9.4 [7.4–11.2]	16.1 [13.2–21.6]	< 0.001
De Backer score (small), n/mm	7.2 [5.7–9.0]	10.3 [7.6–13.9]	< 0.001
PPV (small), %	86.8 [79.5–92.4]	99.3 [98.2–100.0]	< 0.001
MFI (small), AU	2.42 [2.25–2.73]	3.00 [3.00–3.00]	< 0.001

Data are presented as median [IQR]

*TVD* total vascular density, *PVD* perfused vascular density, *PPV* Proportion of perfused vessels, *MFI* microcirculatory flow index

**Table 3:** Differences in microcirculation parameters between survivors and non-survivors

Microcirculation parameter	ICU survivors			*p*	ICU non-survivors			*p*
	Baseline (*n* = 19)	6 h (*n* = 19)	24 h (*n* = 19)		Baseline (*n* = 29)	6 h (*n* = 25)	24 h (*n* = 18)	
Conjunctival								
Conjunctival TVD (small), mm/mm^2^	11.3 [9.2–15.2]	10.6 [9.7–13.6]	11.7 [10.7–13.4]	0.481	12.7 [8.9–14.1]	10.1 [7.5–12.6]	9.7 [7.1–15.0]	0.794
PVD (small), mm/mm^2^	10.0 [8.5–13.7]	10.2 [8.9–12.2]	10.9 [9.6–11.8]	0.257	8.5 [7.0–10.9]	7.6 [5.7–10.3]*	8.3 [5.6–11.4]*	0.735
De Backer score (small), *n*/mm	7.4 [5.8–9.4]	6.7 [5.6–7.9]	7.0 [6.2–8.2]	0.526	7.0 [5.7–8.9]	6.5 [4.5–7.9]	5.9 [4.1–8.8]	0.500
PPV (small), %	91.5 [88.5–93.2]	92.5 [88.4–95.3]	94.4 [88.9–97.5]	0.191	80.6 [70.8–87.3]*	80.8 [70.2–89.3]*	84.1 [72.0–90.1]*	0.735
MFI (small), AU	2.58[ 2.46–2.83]	2.87 [2.64–2.93]^a^	2.92 [2.65–3.00]^a^	0.004	2.25 [1.75–2.50]*	2.25 [1.98–2.59]*	2.50 [2.05–2.59]*	0.640
Retinal								
CRAE, μm	164 [151–189]	166 [152–194]	162 [148–182]	0.344	170 [154–188]	162 [146–175]	175 [147–195]	0.420
CRVE, µm	267 [240–302]	288 [254–306]	266 [236–281]	0.627	245 [223–281]	250 [236–282]*	262 [222–287]	0.627
Vascular length density, %	0.52 [0.43–0.63]	0.55 [0.49–0.60]	0.50 [0.42–0.59]	0.766	0.50 [0.44–0.56]	0.43 [0.40–0.56]*^a^	0.46 [0.39–0.55]^a^	0.188

Data are presented as median [IQR]

*TVD* total vascular density, *PVD* perfused vascular density, *PPV* proportion of perfused vessels, *MFI* microcirculatory flow index, *CRAE* central retinal arteriolar equivalent, *CRVE* central retinal venular equivalent

*p < 0.05 between survivors and non-survivors; ^a^*p* < 0.05 in comparison with baseline

A ROC analysis showed that initial MFI and PPV of small vessels were good predictors of ICU death (AUC = 0.802, *p* = 0.001 and AUC = 0.821, *p* = 0.001, respectively). The optimal cut-off for MFI was 2.33 (sensitivity 94.1%, specificity 65.2%) and for PPV 87.5% (sensitivity 82.4%, specificity 82.6%).

We found no correlations between conjunctival microcirculatory parameters and systemic hemodynamics during all three time points.

### Secondary aim of the study: evaluation of retinal vessels

During the 24-h study period, we did not observe a significant difference between survivors and non-survivors in CRAE. Only at 6 h we observed significant lower CRVE and lower retinal vascular length density in non-survivors compared with survivors (Table 3). Furthermore, non-survivors had significantly lower retinal vascular length density at 6 and 24 h compared to baseline.

### Association between retinal vascular parameters and conjunctival microcirculatory parameters

In septic patients we found no correlations between CRAE, CRVE, vascular length density and conjunctival microcirculatory parameters at baseline and after 24 h (Additional file 4). However, after 6 h we detected significant correlations between TVD, PVD, DeBacker score and CRAE (* r*_s_ = 0.51,* r*_s_ = 0.46, * r*_s_ = 0.46, *p* < 0.05, respectively) as well as between MFI, PPV and vascular length density (* r*_s_ = 0.45, * r*_s_ = 0.56, *p* < 0.05, respectively).

## Discussion

To our knowledge, we are the first to describe changes over time of conjunctival and retinal microvascular parameters in patients with sepsis. First, we found that patients with sepsis had decreased conjunctival microcirculatory parameters in comparison with age-matched healthy controls. This is partly in line with the results from animal studies [13, 26]. In these studies, we and others demonstrated a decreased microvascular flow and reduced perfused small vessel density, but not total small vessel density in the conjunctival microcirculation during very early septic shock. In contrast to animal studies, in this study we found a decrease in total small vessel density in septic patients, suggesting that some vessels did not contain red blood cells. Such difference in the observations of total small vessel density may be explained by the longer lag-time between the onset of sepsis and the start of the observations compared to the animal studies. However, our study lacks a control group such as other critically ill patients also under mechanical ventilation and sedation but without sepsis. Therefore, it is not possible to confirm that changes in total small vessel density are strictly related to a longer lag-time between the onset of sepsis and the time of measurement. Alternatively, changes may be related to other etiologies.

The microcirculation is predominantly studied in the sublingual mucosa using SDF imaging. De Backer [9] with colleagues were the first to demonstrate that all microcirculatory parameters, including total small vessel density, in the sublingual mucosa are decreased in patients with sepsis. In addition, subsequent studies showed that sublingual microcirculatory alterations are more severe in non-survivors than in survivors [10] and that the proportion of perfused small vessels improved over time in response to therapy in survivors but not in non-survivors [12]. Our results in the ocular conjunctiva are partially consistent with those of the published studies in the sublingual mucosa. We found that survivors in comparison with non-survivors had a higher proportion of perfused small vessels, a higher microvascular flow index and a higher perfused small vessels density after 6 and 24 h. In survivors, but not in non-survivors, we observed an increase in microvascular flow index over time. However, we did not observe such difference between survivors and non-survivors in the proportion of perfused small vessels. Such discrepancy may be explained by the fact that microvascular flow index more encompasses convective flow, while the proportion of perfused small vessels is predominantly a marker of diffusion distance. In general, comparison between different vascular beds is more cumbersome in case of parameters of vascular density, but not flow, due to the specific anatomical characteristics of the vascular beds.

Our observations in the conjunctival microcirculation were not matched by the observed absence of significant differences in CRAE between survivors and non-survivors in retinal vessels. We previously reported signs of arteriolar vasodilation with decreased vascular density in septic patients as compared to healthy controls [14]. It is key to understand that retinal fundoscopy enables static detection of blood vessels with larger diameter than capillaries with IDF imaging. This may well explain why the two vascular beds behave differently under conditions of sepsis, despite the fact that changes in density of small conjunctival vessels are mirrored by arteriolar changes in the retina, as expressed by weak but significant correlation coefficients. In this way, retinal vascular length density may not fully represent diffusion capacity.

We did not detect any conjunctival lesions associated with IDF imaging in both patients and healthy volunteers. We did not use a local anesthetic to prevent additional effects on the microcirculation in the conjunctiva. The difficulties of the conjunctival imaging are related to eye movements, patient body movements during mechanical ventilation and the curvature of the ocular wall. We used the patients’ nose as an accessible support for IDF imager but we were not always able to avoid subtle motion artifacts during IDF imaging. In these cases, we reduced motion artifacts during offline video editing. To minimize the mechanical impact of videomicroscopy on the ocular conjunctiva, we reduced the number of repeats to a minimum and shortened the IDF image recording time. We also observed that difficulties in removing pressure artifact are related to the severity of the shock and/or the presence of hypovolemia, or the presence of the dry eye. The eyelids protect the eyes from external influences. Practically conjunctival evaluations may be useful when we are unable to perform a sublingual microcirculatory evaluation, such as in cases of oral trauma or local abscess, or when we have other goals, for example, when evaluating local microcirculation or brain perfusion. However, further studies are needed to determine this. The main disadvantage of conjunctival IDF imaging is the need for good collaboration or sedation.

This study has several other limitations. Our study may have an insufficient sample size to compare differences in the evolution of microvascular changes in conjunctiva and retina over time with clinically relevant endpoint, such as mortality, also due to the considerable number of patients lost to follow-up due to death.

Retinal vessel length density data are dependent on the quality of the retinal photographs and disc centering. However, we made a surplus of images and excluded non-gradable photographs.

We included patients within 24 h from ICU admission and observed the ocular microcirculation three times within 24 h, which may possibly limit the ability to detect all changes in microcirculation. However, in our study median time of patients’ inclusion was 12 h, and previous studies demonstrated that changes affecting mortality occur within the first 24 h from ICU admission [10].

Although we did not observe obvious signs of dry eyes in our patients, we cannot rule out subtle lesions due to hyperemia or swelling. Studies using a functional slit-lamp biomicroscope showed increased blood flow velocity, higher vessel density and larger vessel diameter in the bulbar conjunctiva of patients with dry eyes [27–29]. However, according to the literature most ocular surface disorders in the ICU develop after the inclusion period of the study, i.e., 48–72 h after ICU admission and are often related to lagopthalmos, or incomplete eyelid closure, which was prevented by our daily eye care protocol [30]. This means that the likelihood of ocular surface disorders due to external factors within the first 24 h is low.

The ocular microcirculation was not compared to the sublingual microcirculation and thus it is impossible to determine whether the conjunctival microcirculation is superior to or different from the sublingual microcirculation.

Many studies have been performed with the use of the topical agent tropicamide. However, to our knowledge, we were the first who start the studies using a handheld fundoscope in an ICU environment. Pupil dilation is performed to facilitate better illumination and image capture from the retina. It is very important in ICU environment, where difficulties in retina image registration are due to the patient’s lying position. Furthermore, in the ICU setting it is not possible to create a fully shaded environment and patients are sedated; therefore, they cannot capture the look. Mydriasis with tropicamide is produced within 20 min of instillation and usually lasts for about 6 h. Only few clinical studies were available up to now, investigating the effect of anti-muscarinergic (e.g., tropicamide) agent [31–34]. Tropicamide has been reported to increase retinal arteriolar and venular vasoconstriction and produce a short lasting 30% decrease in parapapillary retinal blood flow [31], although other studies could not detect any changes due to tropicamide in healthy subjects [33, 34]. The effect of tropicamide on the conjunctival microcirculation has not been studied. In our study, we first investigated microcirculation of the conjunctiva and then instillated tropicamide for retinal examination for all patients. Also, the intervals between the eye evaluations are greater than 6 h.

We did one eye evaluation to avoid losing of the relation between the conjunctiva and the retina, if any exist, and for reducing imaging time. However, measurements in both eyes may provide more information about microcirculation of the conjunctiva.

## Conclusions

Microcirculatory perfusion in the conjunctiva was altered in septic patients as compared to healthy controls. Evaluation over 24 h revealed that survivors have a higher microcirculatory flow with an incremental improvement of microvascular flow index over time in comparison with non-survivors. However, we were unable to demonstrate differences in arteriolar retinal vessels between survivors and non-survivors. IDF imaging is feasible tool to assess the microcirculation of the conjunctiva in septic patients.

## Supplementary information

**Additional file 1.** A video clip file showing normal microcirculation in the conjunctiva in a healthy volunteer (2208 × 1648 resolution video file exported from IDF imaging device).

**Additional file 2.** A video clip file showing altered microcirculation in the conjunctiva in a patient with septic shock (reduced resolution (716x572) video file exported for analysis with AVA 3.0).

**Additional file 3.** A video clip file showing altered microcirculation in the conjunctiva in a patient with septic shock (2208x1648 resolution video file exported from IDF imaging device).

**Additional file 4.** Correlations between retinal and conjunctival microcirculatory parameters at baseline and after 24 h.

## Data Availability

The datasets used and/or analyzed during the current study are available from the corresponding author on reasonable request.

## Abbreviations

IDF: Incident dark field illumination
ICU: Intensive care unit
SOFA: Sequential organ failure assessment
MFI: Microvascular flow index
PPV: Proportion of perfused vessels
TVD: Total vessel density
PVD: Perfused vessel density
CRAE: Central retinal arteriolar equivalent
CRVE: Central retinal venular equivalent
APACHE II: Acute physiology and chronic evaluation score
HR: Heart rate
MAP: Mean arterial pressure

## References

[1] Seidelmann SB, Claggett B, Bravo PE, Gupta A, Farhad H, Klein BE, Klein R, Di Carli M, Solomon SD (2016). Retinal vessel calibers in predicting long-term cardiovascular outcomes: the atherosclerosis risk in communities study. Circulation.

[2] Schrijvers EM, Buitendijk GH, Ikram MK, Koudstaal PJ, Hofman A, Vingerling JR, Breteler MM (2012). Retinopathy and risk of dementia: the Rotterdam study. Neurology..

[3] Ponto KA, Werner DJ, Wiedemer L, Laubert-Reh D, Schuster AK, Nickels S, Hohn R, Schulz A, Binder H, Beutel M, Lackner KJ, Wild PS, Pfeiffer N, Mirshahi A (2017). Retinal vessel metrics: normative data and their use in systemic hypertension: results from the Gutenberg Health Study. J Hypertens.

[4] McGeechan K, Liew G, Macaskill P, Irwig L, Klein R, Klein BE, Wang JJ, Mitchell P, Vingerling JR, de Jong PT, Witteman JC, Breteler MM, Shaw J, Zimmet P, Wong TY (2009). Prediction of incident stroke events based on retinal vessel caliber: a systematic review and individual-participant meta-analysis. Am J Epidemiol.

[5] McGeechan K, Liew G, Macaskill P, Irwig L, Klein R, Klein BE, Wang JJ, Mitchell P, Vingerling JR, Dejong PT, Witteman JC, Breteler MM, Shaw J, Zimmet P, Wong TY (2009). Meta-analysis: retinal vessel caliber and risk for coronary heart disease. Ann Intern Med.

[6] Kifley A, Wang JJ, Cugati S, Wong TY, Mitchell P (2007). Retinal vascular caliber, diabetes, and retinopathy. Am J Ophthalmol.

[7] Khansari MM, Tan M, Karamian P, Shahidi M (2018). Inter-visit variability of conjunctival microvascular hemodynamic measurements in healthy and diabetic retinopathy subjects. Microvasc Res.

[8] Hajj J, Blaine N, Salavaci J, Jacoby D (2018). The, “Centrality of sepsis”: a review on incidence, mortality, and cost of care. Healthcare..

[9] De Backer D, Creteur J, Preiser JC, Dubois MJ, Vincent JL (2002). Microvascular blood flow is altered in patients with sepsis. Am J Respir Crit Care Med.

[10] De Backer D, Donadello K, Sakr Y, Ospina-Tascon G, Salgado D, Scolletta S, Vincent JL (2013). Microcirculatory alterations in patients with severe sepsis: impact of time of assessment and relationship with outcome. Crit Care Med.

[11] Hernandez G, Boerma EC, Dubin A, Bruhn A, Koopmans M, Edul VK, Ruiz C, Castro R, Pozo MO, Pedreros C, Veas E, Fuentealba A, Kattan E, Rovegno M, Ince C (2013). Severe abnormalities in microvascular perfused vessel density are associated to organ dysfunctions and mortality and can be predicted by hyperlactatemia and norepinephrine requirements in septic shock patients. J Crit Care..

[12] Sakr Y, Dubois MJ, De Backer D, Creteur J, Vincent JL (2004). Persistent microcirculatory alterations are associated with organ failure and death in patients with septic shock. Crit Care Med.

[13] Pranskunas A, Pilvinis V, Dambrauskas Z, Rasimaviciute R, Planciuniene R, Dobozinskas P, Veikutis V, Vaitkaitis D, Boerma EC (2012). Early course of microcirculatory perfusion in eye and digestive tract during hypodynamic sepsis. Crit Care.

[14] Simkiene J, Pranskuniene Z, Patasius M, Trumpaitis J, Boerma EC, Pranskunas A (2019). Alterations of retinal vessels in patients with sepsis. J Clin Monit Comput..

[15] Singer M, Deutschman CS, Seymour CW, Shankar-Hari M, Annane D, Bauer M, Bellomo R, Bernard GR, Chiche JD, Coopersmith CM, Hotchkiss RS, Levy MM, Marshall JC, Martin GS, Opal SM, Rubenfeld GD, van der Poll T, Vincent JL, Angus DC (2016). The third international consensus definitions for sepsis and septic shock (Sepsis-3). JAMA.

[16] Rhodes A, Evans LE, Alhazzani W, Levy MM, Antonelli M, Ferrer R, Kumar A, Sevransky JE, Sprung CL, Nunnally ME, Rochwerg B, Rubenfeld GD, Angus DC, Annane D, Beale RJ, Bellinghan GJ, Bernard GR, Chiche JD, Coopersmith C, De Backer DP, French CJ, Fujishima S, Gerlach H, Hidalgo JL, Hollenberg SM, Jones AE, Karnad DR, Kleinpell RM, Koh Y, Lisboa TC, Machado FR, Marini JJ, Marshall JC, Mazuski JE, McIntyre LA, McLean AS, Mehta S, Moreno RP, Myburgh J, Navalesi P, Nishida O, Osborn TM, Perner A, Plunkett CM, Ranieri M, Schorr CA, Seckel MA, Seymour CW, Shieh L, Shukri KA, Simpson SQ, Singer M, Thompson BT, Townsend SR, Van der Poll T, Vincent JL, Wiersinga WJ, Zimmerman JL, Dellinger RP (2017). Surviving sepsis campaign: international guidelines for management of sepsis and septic shock: 2016. Intensive Care Med.

[17] Aykut G, Veenstra G, Scorcella C, Ince C, Boerma C (2015). Cytocam-IDF (incident dark field illumination) imaging for bedside monitoring of the microcirculation. Intensive Care Med Exp..

[18] van Zijderveld R, Ince C, Schlingemann RO (2014). Orthogonal polarization spectral imaging of conjunctival microcirculation. Graefes Arch Clin Exp Ophthalmol.

[19] Pranskunas A, Tamosuitis T, Balciuniene N, Damanskyte D, Sneider E, Vitkauskiene A, Sirvinskas E, Pilvinis V, Boerma EC (2018). Alterations of conjunctival glycocalyx and microcirculation in non-septic critically ill patients. Microvasc Res.

[20] Ince C, Boerma EC, Cecconi M, De Backer D, Shapiro NI, Duranteau J, Pinsky MR, Artigas A, Teboul JL, Reiss IKM, Aldecoa C, Hutchings SD, Donati A, Maggiorini M, Taccone FS, Hernandez G, Payen D, Tibboel D, Martin DS, Zarbock A, Monnet X, Dubin A, Bakker J, Vincent JL, Scheeren TWL, Cardiovascular Dynamics Section of the ESICM (2018). Second consensus on the assessment of sublingual microcirculation in critically ill patients: results from a task force of the European society of intensive care medicine. Intensive Care Med..

[21] Dobbe JG, Streekstra GJ, Atasever B, van Zijderveld R, Ince C (2008). Measurement of functional microcirculatory geometry and velocity distributions using automated image analysis. Med Biol Eng Comput..

[22] Boerma EC, Mathura KR, van der Voort PH, Spronk PE, Ince C (2005). Quantifying bedside-derived imaging of microcirculatory abnormalities in septic patients: a prospective validation study. Crit Care.

[23] Bankhead P, Scholfield CN, McGeown JG, Curtis TM (2012). Fast retinal vessel detection and measurement using wavelets and edge location refinement. PLoS ONE.

[24] Pilat Anastasia V, Proudlock Frank A, McLean Rebecca J, Lawden Mark C, Gottlob Irene (2014). Morphology of retinal vessels in patients with optic nerve head drusen and optic disc edema. Invest Ophthalmol Vis Sci.

[25] Knudtson MD, Lee KE, Hubbard LD, Wong TY, Klein R, Klein BE (2003). Revised formulas for summarizing retinal vessel diameters. Curr Eye Res.

[26] Hessler M, Arnemann PH, Zamit F, Seidel L, Kampmeier TG, Kathofer U, Alnawaiseh M, Tchaichian S, Rehberg S, Ertmer C (2019). Monitoring of conjunctival microcirculation reflects sublingual microcirculation in ovine septic and hemorrhagic shock. Shock..

[27] Alansari MA, Hijazi MH, Maghrabi KA (2015). Making a difference in eye care of the critically Ill patients. J Intensive Care Med..

[28] de Araujo DD, Almeida NG, Silva PM, Ribeiro NS, Werli-Alvarenga A, Chianca TC (2016). Prediction of risk and incidence of dry eye in critical patients. Rev Lat Am Enfermagem..

[29] Grixti A, Sadri M, Edgar J, Datta AV (2012). Common ocular surface disorders in patients in intensive care units. Ocul Surf..

[30] Marshall AP, Elliott R, Rolls K, Schacht S, Boyle M (2008). Eyecare in the critically ill: clinical practice guideline. Aust Crit Care..

[31] Harazny JM, Schmieder RE, Welzenbach J, Michelson G (2013). Local application of tropicamide 0.5% reduces retinal capillary blood flow. Blood Press..

[32] Tsui E, Sehi M, Cheng RW, Wan J, Wong T, Dorner S, Fisher JA, Hudson C (2013). The impact of topical mydriatic ophthalmic solutions on retinal vascular reactivity and blood flow. Exp Eye Res.

[33] Hohberger B, Muller M, Hosari S, Mardin CY (2019). OCT-angiography: mydriatic phenylephrine and tropicamide do not influence retinal microvasculature in macula and peripapillary region. PLoS One..

[34] Frost S, Gregory C, Robinson L, Yu S, Xiao D, Mehdizadeh M, Burnham S, Dehghani C, Vignarajan J, Kanagasingam Y, Schlaich MP, Prentice D (2019). Effect of pupil dilation with tropicamide on retinal vascular caliber. Ophthalmic Epidemiol.

