# Supplementary material for: Ocular microvascular changes in patients with sepsis: a prospective observational study
Source: Ann Intensive Care. 2020 Apr 7;10:38. doi: 10.1186/s13613-020-00655-x (PMC7138894; doi:10.1186/s13613-020-00655-x)
Supplement: Supplementary file 4 — Additional file 4. Correlations between retinal and conjunctival microcirculatory parameters at baseline and after 24 h. [file 13613_2020_655_MOESM4_ESM.docx]

**Ocular microvascular changes in patients with sepsis: a prospective observational study**

**Online Supplemental Content**

**Supplemental Table 1**. Correlations between the central retinal arteriolar equivalent (CRAE) and conjunctival microcirculatory variables

|  | **Baseline** | | **After 24 hours** | |
| --- | --- | --- | --- | --- |
|  | **Spearman’s Rho** | ***p* value** | **Spearman’s Rho** | ***p* value** |
| Total small vessel density | 0.09 | 0.585 | -0.04 | 0.856 |
| Perfused small vessel density | 0.04 | 0.792 | 0.01 | 0.947 |
| Proportion of perfused small vessels | 0.18 | 0.263 | 0.15 | 0.454 |
| Microvascular Flow Index | 0.12 | 0.487 | 0.07 | 0.742 |
| De Backer score | 0.13 | 0.442 | -0.13 | 0.508 |

**Supplemental Table 2**. Correlations between the central retinal venular equivalent (CRVE) and conjunctival microcirculatory variables

|  | **Baseline** | | **After 24 hours** | |
| --- | --- | --- | --- | --- |
|  | **Spearman’s Rho** | ***p* value** | **Spearman’s Rho** | ***p* value** |
| Total small vessel density | 0.10 | 0.539 | 0.22 | 0.264 |
| Perfused small vessel density | 0.08 | 0.631 | 0.25 | 0.197 |
| Proportion of perfused small vessels | 0.24 | 0.141 | 0.04 | 0.829 |
| Microvascular Flow Index | 0.17 | 0.312 | 0.04 | 0.844 |
| De Backer score | 0.08 | 0.623 | 0.20 | 0.318 |

**Supplemental Table 3**. Correlations between the retinal vascular length density and conjunctival microcirculatory variables

|  | **Baseline** | | **After 24 hours** | |
| --- | --- | --- | --- | --- |
|  | **Spearman’s Rho** | ***p* value** | **Spearman’s Rho** | ***p* value** |
| Total small vessel density | -0.08 | 0.605 | 0.14 | 0.502 |
| Perfused small vessel density | -0.04 | 0.819 | 0.22 | 0.275 |
| Proportion of perfused small vessels | 0.14 | 0.376 | 0.22 | 0.261 |
| Microvascular Flow Index | 0.21 | 0.199 | 0.28 | 0.157 |
| De Backer score | -0.14 | 0.393 | 0.13 | 0.534 |
